# Supplementary material for: A Likelihood Approach for Real-Time Calibration of Stochastic Compartmental Epidemic Models
Source: PLoS Comput Biol. 2017 Jan 17;13(1):e1005257. doi: 10.1371/journal.pcbi.1005257 (PMC5240920; doi:10.1371/journal.pcbi.1005257)
Supplement: S1 File — (TAR.GZ) [file pcbi.1005257.s014.tar.gz › HSPH_Online-SI-Revision/EnKF07/n100-severe/n100-severe_EnKF_table4.pdf]

1	R0	{1.65278, 9.06793}	{2.49168, 3.71699}	{{1.45809, 2.91278}, {3., 5.10673}}	{58.4697, 60.728}	inf	4608.	11 180.6	{2090.36, 45 991.7}	165.674	0.292763	newly	3152.	14 554.	{2615., 48 461.}	371.122	0.492308	Reff	0.666979	0.657273	{0.232027, 1.11788}	33.2441	ar	66 342.	192 144.	{163 174., 283 113.}	189.626
2	R0	{1.4321, 3.80644}	{2.59846, 5.92199}	{{1.68976, 2.9249}, {3., 7.17747}}	{81.7553, 66.6926}	inf	684.	3072.65	{209.064, 4782.26}	384.01	0.535505	newly	952.	3236.23	{151., 5769.}	289.812	0.533601	Reff	0.675178	0.496235	{0.14214, 0.807384}	45.1172	ar	54 217.	173 706.	{162 654., 188 784.}	220.391
3	R0	{1.68388, 4.76028}	{2.45101, 10.6261}	{{1.34829, 8.40089}, {3., 14.7848}}	{51.6207, 123.224}	inf	788.	7130.46	{636.053, 7184.58}	816.875	0.747887	newly	834.	4526.07	{461., 7690.}	463.215	0.567762	Reff	0.549854	0.598939	{0.160537, 1.53565}	76.7591	ar	68 299.	194 642.	{170 887., 259 396.}	184.985
4	R0	{1.66014, 6.95383}	{2.22746, 7.37791}	{{0.774894, 5.64301}, {3., 8.21079}}	{70.7469, 17.982}	inf	3183.	5001.03	{1089.79, 7049.92}	132.371	0.346224	newly	2735.	3592.2	{744., 5600.}	123.15	0.40553	Reff	0.623814	0.378848	{0.104808, 0.602982}	43.0898	ar	66 820.	170 170.	{158 324., 182 369.}	154.669
5	R0	{1.69984, 11.2038}	{2.51143, 3.43571}	{{1.56173, 2.77308}, {3., 5.10683}}	{53.2182, 69.3345}	inf	5824.	12 048.4	{2747.51, 43 838.4}	134.76	0.257771	newly	3233.	13 319.	{3408., 43 252.}	320.699	0.444249	Reff	0.680072	0.510165	{0.139483, 0.888038}	39.0027	ar	68 462.	185 968.	{162 533., 273 556.}	171.637
6	R0	{1.65876, 6.66217}	{2.19543, 7.35654}	{{0.763577, 5.54595}, {3., 8.04503}}	{69.5275, 19.135}	inf	3387.	4437.83	{1385.64, 4186.65}	98.9138	0.308223	newly	2924.	3316.23	{1005., 3950.}	99.8861	0.380946	Reff	0.632386	0.406051	{0.0374307, 0.599115}	46.58	ar	67 105.	167 430.	{155 053., 177 218.}	149.504
7	R0	{1.69966, 5.89996}	{2.42089, 5.35511}	{{1.4068, 2.53263}, {3., 6.89321}}	{47.8429, 29.7438}	inf	2191.	4725.51	{481.361, 3885.79}	166.491	0.349213	newly	2014.	5565.54	{392., 5331.}	228.139	0.385227	Reff	0.564848	0.43746	{0.153353, 0.696887}	37.3226	ar	69 396.	177 770.	{164 724., 186 579.}	156.167
8	R0	{1.53428, 6.3734}	{2.2865, 3.29432}	{{1.17928, 1.7455}, {3., 4.98052}}	{69.4661, 48.3114}	inf	2878.	2156.87	{534.269, 3059.87}	89.6094	0.491378	newly	2677.	3738.52	{496., 3293.}	124.189	0.390053	Reff	0.700629	0.545431	{0.223467, 0.956886}	42.0417	ar	59 855.	175 516.	{163 398., 199 151.}	193.236
9	R0	{1.58939, 3.48608}	{2.53482, 10.5778}	{{1.90868, 0.29417}, {3., 19.3511}}	{59.4835, 241.835}	inf	666.	12 803.9	{1712.39, 29 239.2}	1830.68	0.981973	newly	876.	13 956.6	{733., 35 099.}	1505.07	0.829335	Reff	0.606066	0.896697	{0.240978, 1.88449}	78.9574	ar	62 575.	211 645.	{172 010., 293 968.}	238.227
10	R0	{1.58528, 5.5593}	{2.65551, 3.26153}	{{2.01574, 2.35733}, {3., 4.05316}}	{67.5104, 41.332}	inf	2018.	10 937.6	{1855.65, 26 256.5}	447.588	0.495933	newly	2025.	22 004.1	{2989., 72 040.}	988.796	0.769083	Reff	0.636823	1.05781	{0.533607, 2.01572}	76.6611	ar	63 039.	202 660.	{166 458., 277 071.}	221.483
11	R0	{1.58305, 4.62721}	{2.39158, 5.29593}	{{1.37784, 2.56259}, {3., 6.91444}}	{54.8751, 44.5502}	inf	1344.	4907.99	{330.797, 4145.91}	295.615	0.434685	newly	1559.	6083.25	{221., 5936.}	331.096	0.466395	Reff	0.61568	0.459462	{0.184966, 0.742805}	39.1635	ar	63 222.	178 622.	{164 372., 196 160.}	182.532
12	R0	{1.70234, 9.34626}	{2.41849, 3.60921}	{{1.46838, 2.86867}, {3., 5.13421}}	{52.4539, 62.9664}	inf	5033.	10 094.7	{1011.95, 44 134.6}	127.044	0.290978	newly	3340.	12 208.1	{1081., 48 020.}	284.051	0.450932	Reff	0.662312	0.472274	{0.136627, 1.14219}	54.8115	ar	68 213.	186 916.	{165 293., 277 617.}	174.018
13	R0	{1.68556, 9.74954}	{2.47773, 3.62204}	{{1.46247, 2.93221}, {3., 5.0844}}	{55.9393, 64.3014}	inf	4862.	13 592.3	{2774.29, 46 914.6}	199.307	0.304891	newly	3072.	15 557.2	{4801., 55 790.}	413.151	0.535534	Reff	0.650053	0.590333	{0.26789, 0.907618}	32.1944	ar	68 426.	192 322.	{160 688., 278 565.}	181.066
14	R0	{1.59876, 9.78897}	{2.47469, 3.43872}	{{1.49755, 2.81837}, {3., 5.1399}}	{59.1511, 64.8715}	inf	5531.	10 817.2	{3260.51, 43 622.1}	112.021	0.213656	newly	3640.	13 964.5	{4622., 47 109.}	289.496	0.447662	Reff	0.761314	0.60763	{0.285163, 1.09459}	38.4949	ar	63 553.	191 805.	{169 756., 278 884.}	201.803
15	R0	{1.43577, 5.12819}	{2.1252, 10.3829}	{{0.513442, 6.17922}, {3., 18.8799}}	{91.4345, 102.467}	inf	2030.	5456.76	{625.967, 11 891.9}	203.341	0.424325	newly	2303.	4180.83	{240., 7284.}	146.218	0.470765	Reff	0.736378	0.612075	{0.157339, 0.896963}	28.5962	ar	53 259.	170 676.	{157 692., 182 527.}	220.465
16	R0	{1.58866, 8.73191}	{2.66675, 2.95878}	{{2.07431, 1.09276}, {3., 3.99049}}	{67.8617, 66.1153}	inf	5612.	11 611.6	{3051.66, 30 424.9}	128.119	0.259871	newly	4036.	23 290.4	{3951., 59 038.}	481.652	0.537082	Reff	0.723984	0.93292	{0.293891, 2.07429}	57.8382	ar	64 349.	205 292.	{170 107., 266 060.}	219.029
17	R0	{1.43012, 3.8163}	{2.42152, 5.52602}	{{1.69541, 2.64632}, {3., 7.196}}	{69.3226, 64.7684}	inf	1091.	4602.21	{132.605, 5283.68}	357.364	0.521127	newly	1516.	6833.92	{79., 6262.}	397.485	0.522058	Reff	0.70422	0.543585	{0.141519, 1.01616}	44.5158	ar	53 353.	175 605.	{159 832., 206 954.}	229.138
18	R0	{1.47965, 5.16472}	{2.62066, 7.87691}	{{2.22933, 3.70874}, {3., 9.34217}}	{83.7654, 67.3535}	inf	1756.	5072.79	{530.271, 10 387.5}	226.368	0.398228	newly	1958.	4486.43	{330., 12 669.}	184.888	0.406869	Reff	0.705823	0.613865	{0.120471, 1.66487}	71.939	ar	56 086.	179 049.	{158 459., 237 428.}	219.241
19	R0	{1.57191, 5.2314}	{2.63312, 7.27133}	{{2.16106, 3.58266}, {3., 8.81305}}	{71.0617, 56.5971}	inf	1293.	3823.67	{253.009, 6235.19}	259.63	0.465666	newly	1298.	3730.88	{172., 7965.}	269.19	0.561034	Reff	0.610687	0.575143	{0.153832, 1.39747}	70.6861	ar	63 119.	170 121.	{153 844., 198 515.}	169.524
20	R0	{1.54435, 5.32911}	{2.33533, 8.18529}	{{1.17553, 3.53306}, {3., 13.6817}}	{71.1647, 63.7067}	inf	1762.	4006.57	{492.916, 5596.17}	166.271	0.407708	newly	1849.	3279.53	{197., 6913.}	123.822	0.401648	Reff	0.632813	0.560722	{0.0893953, 1.17388}	42.55	ar	61 870.	173 984.	{161 146., 206 231.}	181.208
21	R0	{1.57932, 4.90031}	{2.3849, 5.40457}	{{1.52665, 2.56465}, {3., 6.96183}}	{52.3406, 41.5289}	inf	1650.	4803.85	{512.475, 4077.73}	222.207	0.34729	newly	1742.	5781.01	{373., 6780.}	273.75	0.405282	Reff	0.608496	0.438952	{0.114992, 0.781493}	41.7044	ar	63 595.	176 193.	{163 104., 199 118.}	177.054
22	R0	{1.47044, 4.07694}	{2.54722, 9.97535}	{{1.14801, 4.4955}, {3., 13.9953}}	{81.1586, 144.677}	inf	1362.	3172.97	{763.306, 3386.65}	158.903	0.246822	newly	1880.	2489.86	{344., 3837.}	118.034	0.401917	Reff	0.691357	0.662495	{0.165555, 1.84799}	71.9228	ar	55 895.	171 813.	{154 478., 224 256.}	207.386
23	R0	{1.64997, 3.34546}	{2.30734, 5.58684}	{{1.90968, 1.14749}, {3., 15.3162}}	{53.5653, 148.162}	inf	230.	9333.26	{1185.49, 22 148.6}	3958.16	1.18417	newly	308.	10 479.9	{923., 34 615.}	3303.23	1.11528	Reff	0.558729	0.538251	{0.110333, 1.20333}	60.7538	ar	66 404.	183 819.	{160 239., 268 233.}	176.819
24	R0	{1.67405, 4.50254}	{1.97455, 6.17031}	{{0.63014, 3.7076}, {3., 7.83427}}	{67.5923, 53.2837}	inf	1189.	3667.1	{1178.29, 2892.15}	217.762	0.408834	newly	1389.	3079.71	{939., 3552.}	134.741	0.285279	Reff	0.560623	0.328505	{0.105641, 0.514466}	42.5896	ar	68 017.	173 797.	{165 202., 170 825.}	155.52
25	R0	{1.50663, 3.1286}	{1.92673, 6.77417}	{{0.673676, 3.69468}, {3., 8.88139}}	{71.9369, 116.524}	inf	450.	4064.34	{1368.17, 3396.93}	803.187	0.809451	newly	698.	3121.41	{1088., 4371.}	349.64	0.525497	Reff	0.634201	0.347475	{0.125604, 0.584501}	45.9441	ar	58 823.	168 974.	{161 553., 185 811.}	187.258
26	R0	{1.69822, 6.34992}	{2.67518, 11.7727}	{{1.88376, 3.50333}, {3., 19.7438}}	{57.5285, 116.274}	inf	2723.	3084.66	{467.634, 6503.03}	88.0589	0.406233	newly	2372.	3065.61	{198., 6892.}	128.304	0.583181	Reff	0.56493	0.708871	{0.117992, 1.80493}	62.4394	ar	69 816.	171 910.	{157 911., 230 001.}	146.233
27	R0	{1.55869, 4.92988}	{2.49495, 7.25079}	{{0.748647, 3.53003}, {3., 8.92338}}	{75.7924, 64.0527}	inf	1548.	5338.88	{210.5, 3296.67}	302.608	0.492953	newly	1681.	4864.82	{173., 6333.}	263.333	0.537286	Reff	0.625643	0.540435	{0.0808849, 1.1281}	60.764	ar	62 344.	167 910.	{151 411., 191 017.}	169.329
28	R0	{1.46085, 6.06899}	{2.59616, 12.0234}	{{1.90553, 5.28385}, {3., 15.723}}	{86.5545, 104.013}	inf	2116.	3553.01	{324.502, 4232.59}	120.234	0.394741	newly	2108.	2292.32	{265., 2721.}	97.5655	0.470619	Reff	0.722157	0.63899	{0.0251913, 1.7337}	62.4188	ar	55 484.	174 586.	{158 195., 206 316.}	214.66
29	R0	{1.63017, 6.25164}	{2.63872, 7.65898}	{{2.18062, 3.55975}, {3., 8.75715}}	{66.9714, 39.7244}	inf	2357.	2282.23	{253.886, 2516.29}	79.0531	0.425013	newly	2132.	2125.16	{169., 3495.}	94.6857	0.528368	Reff	0.606896	0.695926	{0.0986817, 1.54935}	57.6022	ar	66 231.	169 697.	{155 221., 217 596.}	156.22
30	R0	{1.45173, 5.68165}	{2.70379, 3.23645}	{{2.07436, 2.2735}, {3., 3.99044}}	{86.2463, 43.0367}	inf	2119.	9281.76	{1577.5, 16 282.5}	348.877	0.438814	newly	2202.	19 955.2	{2237., 62 721.}	812.078	0.691742	Reff	0.721074	1.06031	{0.638408, 2.07434}	57.6116	ar	55 280.	193 740.	{160 378., 268 523.}	250.47
31	R0	{1.55808, 2.77316}	{2.00531, 6.03508}	{{0.377446, 3.65727}, {3., 8.06152}}	{86.0513, 117.625}	inf	226.	2604.32	{890.638, 2869.68}	1054.27	0.986134	newly	342.	2033.27	{1004., 3539.}	496.506	0.733249	Reff	0.613478	0.311187	{0.065271, 0.523776}	49.275	ar	60 940.	172 542.	{168 764., 173 370.}	183.134
32	R0	{1.56797, 3.90327}	{2.70366, 4.45112}	{{2.23892, 3.81435}, {3., 4.83359}}	{72.4307, 18.3791}	inf	914.	6227.5	{4627.77, 5886.44}	584.356	0.813357	newly	1187.	9028.72	{4875., 19 303.}	663.675	0.793892	Reff	0.61673	0.69616	{0.229887, 1.73526}	59.7141	ar	62 228.	197 040.	{174 059., 264 163.}	216.643
33	R0	{1.70027, 5.43261}	{2.57597, 4.52261}	{{1.88413, 4.19398}, {3., 4.58578}}	{51.5037, 21.0113}	inf	1215.	5143.23	{2922.93, 4309.51}	331.322	0.57845	newly	1142.	6401.19	{3382., 6028.}	467.067	0.609275	Reff	0.555682	0.46437	{0.0670674, 0.982433}	62.7868	ar	68 798.	185 125.	{170 426., 220 395.}	169.085
34	R0	{1.61668, 6.86192}	{2.43084, 3.08459}	{{1.4584, 1.15071}, {3., 5.00576}}	{57.4526, 55.0477}	inf	2934.	13 030.8	{4062.44, 42 936.8}	348.769	0.506853	newly	2525.	16 723.5	{5798., 46 353.}	563.766	0.691842	Reff	0.646575	0.612644	{0.0705033, 1.256}	52.5006	ar	64 693.	209 199.	{180 072., 284 012.}	223.372
35	R0	{1.55955, 3.45071}	{2.42066, 5.89537}	{{1.91471, 1.57199}, {3., 16.3547}}	{65.5388, 131.085}	inf	514.	5859.49	{1749.84, 6960.34}	1042.22	0.861885	newly	674.	8887.52	{1308., 12 451.}	1220.13	0.803991	Reff	0.608474	0.562778	{0.158257, 1.1802}	56.1861	ar	61 783.	183 119.	{165 787., 242 749.}	196.391
36	R0	{1.65068, 4.91307}	{2.39161, 5.32627}	{{1.59404, 2.40524}, {3., 6.93821}}	{46.29, 41.8111}	inf	1866.	4652.13	{287.31, 3929.16}	199.827	0.398862	newly	2044.	5610.79	{240., 6112.}	234.668	0.450595	Reff	0.596341	0.409082	{0.0817104, 0.679553}	44.1318	ar	66 453.	177 633.	{163 602., 193 236.}	167.306
37	R0	{1.46131, 3.06714}	{2.45865, 5.50822}	{{1.96195, 1.50995}, {3., 17.0375}}	{77.8744, 139.044}	inf	344.	6141.29	{2076.18, 10 130.1}	1686.77	1.01304	newly	544.	8915.95	{973., 14 226.}	1540.98	0.888863	Reff	0.646659	0.535526	{0.17902, 0.684664}	46.7866	ar	56 452.	174 387.	{157 401., 225 679.}	208.911
38	R0	{1.71034, 5.2546}	{2.53843, 5.15665}	{{1.56141, 2.95808}, {3., 6.07783}}	{54.1323, 22.6933}	inf	1519.	5609.71	{394.266, 14 050.1}	296.889	0.476566	newly	1497.	7935.45	{362., 34 230.}	460.162	0.511543	Reff	0.547719	0.546891	{0.241174, 1.03156}	47.9636	ar	69 816.	186 315.	{168 247., 233 897.}	166.866
39	R0	{1.43565, 5.57989}	{2.53843, 12.5566}	{{1.90398, 5.4203}, {3., 15.751}}	{87.2316, 126.043}	inf	1796.	3899.61	{385.559, 5239.84}	169.526	0.416014	newly	1875.	2781.44	{192., 2853.}	135.007	0.490063	Reff	0.709096	0.55907	{0.0286849, 1.36758}	58.8182	ar	54 407.	174 085.	{159 094., 212 699.}	219.968
40	R0	{1.40327, 4.00764}	{2.4959, 5.95652}	{{1.70445, 3.02724}, {3., 7.49873}}	{77.8634, 61.7625}	inf	1177.	3049.48	{366.715, 5316.89}	200.587	0.396583	newly	1644.	4112.17	{251., 6085.}	210.184	0.431341	Reff	0.717576	0.511651	{0.257069, 0.801992}	43.0714	ar	51 976.	169 904.	{158 856., 181 499.}	226.89
41	R0	{1.63612, 4.26741}	{2.43518, 5.93891}	{{1.93746, 1.6473}, {3., 16.9597}}	{60.7525, 121.433}	inf	805.	5876.93	{1580.11, 11 162.6}	633.54	0.658773	newly	987.	7894.3	{1349., 13 983.}	703.145	0.612795	Reff	0.590394	0.601151	{0.153122, 1.58809}	64.4368	ar	65 029.	180 241.	{159 836., 242 435.}	177.171
42	R0	{1.57426, 6.50271}	{2.14821, 10.2704}	{{0.443831, 6.27047}, {3., 18.7972}}	{81.277, 59.5697}	inf	2814.	6875.32	{1181.73, 13 670.8}	170.556	0.359113	newly	2604.	5211.7	{447., 7508.}	145.287	0.39991	Reff	0.666321	0.657416	{0.149621, 0.808102}	31.4697	ar	62 522.	178 390.	{161 452., 194 974.}	185.324
43	R0	{1.37818, 5.37636}	{2.70938, 3.19785}	{{2.09181, 2.24488}, {3., 3.97176}}	{96.5912, 40.5202}	inf	1811.	7767.16	{1683.39, 18 519.3}	333.284	0.477901	newly	2047.	18 446.7	{2062., 62 588.}	803.484	0.664201	Reff	0.756841	0.955496	{0.408597, 2.09179}	54.2183	ar	50 098.	190 196.	{160 302., 266 254.}	279.648
44	R0	{1.66606, 4.92641}	{2.69143, 5.62568}	{{2.19393, 0.431711}, {3., 7.54734}}	{61.5444, 50.7362}	inf	1739.	7756.11	{2356.83, 7051.37}	351.051	0.578516	newly	1807.	8810.62	{2000., 17 045.}	394.991	0.594249	Reff	0.583371	0.711812	{0.211824, 1.4669}	54.8909	ar	67 066.	197 616.	{172 463., 258 379.}	194.659
45	R0	{1.623, 5.9449}	{2.22133, 7.3585}	{{0.749272, 5.52735}, {3., 7.98965}}	{71.733, 27.1496}	inf	2642.	4662.75	{2161.72, 5466.83}	107.477	0.178237	newly	2549.	3471.77	{1412., 4239.}	103.095	0.276501	Reff	0.641799	0.413573	{0.123202, 0.563854}	43.3201	ar	64 554.	168 971.	{157 972., 180 767.}	161.752
46	R0	{1.65235, 5.51234}	{2.5258, 7.37974}	{{0.748954, 3.50519}, {3., 8.91697}}	{67.8701, 53.4638}	inf	2356.	2545.12	{326.543, 5100.23}	81.2396	0.374463	newly	2372.	2394.32	{222., 6796.}	96.6332	0.526517	Reff	0.608825	0.511445	{0.0808613, 1.59182}	78.918	ar	66 509.	163 475.	{148 808., 229 411.}	145.794
47	R0	{1.62138, 8.82208}	{2.76058, 3.08243}	{{2.02026, 3.04119}, {3., 3.16492}}	{70.261, 65.0601}	inf	4208.	14 801.1	{8832.29, 13 362.6}	253.065	0.499079	newly	2927.	26 422.2	{12 501., 52 540.}	802.705	0.867356	Reff	0.666095	0.868763	{0.135458, 2.01944}	72.8792	ar	65 598.	201 651.	{170 549., 255 414.}	207.404
48	R0	{1.64073, 6.69261}	{2.20767, 7.30038}	{{0.768923, 5.59922}, {3., 8.09444}}	{71.4881, 19.2739}	inf	3295.	4273.75	{375.001, 4081.58}	105.997	0.388313	newly	2847.	3228.54	{261., 3405.}	106.146	0.45721	Reff	0.626628	0.415483	{0.0995252, 0.685467}	46.3784	ar	66 438.	163 184.	{152 643., 166 458.}	145.618
49	R0	{1.65556, 3.796}	{2.03604, 5.77499}	{{0.640978, 3.70507}, {3., 7.84492}}	{67.9707, 54.5291}	inf	539.	3065.68	{1846., 2896.65}	469.692	0.703637	newly	685.	2654.11	{1772., 3623.}	289.12	0.542097	Reff	0.564513	0.363259	{0.117017, 0.597121}	38.6711	ar	66 575.	168 981.	{162 379., 186 717.}	153.82
50	R0	{1.56828, 3.65713}	{2.42565, 6.02198}	{{1.94206, 1.58564}, {3., 16.7713}}	{63.4696, 136.132}	inf	506.	5102.83	{2107.34, 4366.92}	909.272	0.818573	newly	685.	8890.35	{1039., 24 791.}	1199.32	0.763394	Reff	0.595868	0.592988	{0.158873, 1.21071}	61.2636	ar	62 761.	178 386.	{160 197., 240 574.}	184.23
